# Characteristics of rheumatoid arthritis and its association with major comorbid conditions: cross-sectional study of 502 649 UK Biobank participants

**DOI:** 10.1136/rmdopen-2016-000267

**Published:** 2016-06-14

**Authors:** Stefan Siebert, Donald M Lyall, Daniel F Mackay, Duncan Porter, Iain B McInnes, Naveed Sattar, Jill P Pell

**Affiliations:** 1 Institute of Infection, Immunity and Inflammation, University of Glasgow, Glasgow, UK; 2 Institute of Health & Wellbeing, University of Glasgow, Glasgow, UK; 3 Institute of Cardiovascular and Medical Sciences, University of Glasgow, Glasgow, UK

**Keywords:** DMARDs (biologic), DMARDs (synthetic), Rheumatoid Arthritis, Epidemiology

## Abstract

**Introduction:**

To characterise the detailed phenotypic and comorbid characteristics of participants with rheumatoid arthritis (RA) in the large population-based UK Biobank, thereby enabling future longitudinal analyses.

**Methods:**

We undertook a cross-sectional study using baseline data from the unique UK Biobank resource (n=502 649). RA was based on self-report, and type of medication was used as a proxy measure of valid diagnosis. Participants with and without RA were compared in terms of sociodemographic, lifestyle and other disease-related risk factors. Logistic regression models were used to determine whether participants with RA were more likely to report comorbid conditions, and whether this varied by RA severity. The models were adjusted for potential confounders and lifestyle risk factors.

**Results:**

At baseline, 5657 (1.13%) eligible UK Biobank participants reported RA of whom 2849 (0.57%) had medically treated RA (median duration=10 years). Prevalence was significantly higher among female, South Asian and socioeconomically deprived participants. Participants with RA were significantly more likely to report diabetes (covariate-adjusted OR 1.18, 95% CI 1.06 to 1.32, p<0.01), hypertension (OR 1.19, 95% CI 1.21 to 1.27, p<0.001) and cardiovascular disease (OR 1.52, 95% CI 1.39 to 1.67, p<0.001).

**Conclusions:**

UK Biobank provides extensive data concerning RA population-level comorbidity and risk factors. The frequency, distribution and characteristics of participants reporting RA in UK Biobank are largely consistent with other studies. It provides a unique opportunity to interrogate biomarkers, genetic data, detailed imaging and linkage to clinical records at the population level across primary and secondary care.

**Key messages**

**What is already known about this subject?**

- Rheumatoid arthritis (RA) is associated with significant cardiometabolic and psychiatric comorbidities that require long-term studies to evaluate.
- UK Biobank is a large population-based cohort study that offers opportunities to study chronic diseases.

**What does this study add?**

- This is the first report of the baseline characteristics of the RA population in UK Biobank.
- The prevalence of RA in UK Biobank is 1.13% by self-report and 0.57% by medication.
- Patients with RA were significantly more likely to report diabetes, hypertension and cardiovascular disease.

**How might this impact on clinical practice?**

- UK Biobank emphasises the burden of RA and comorbidities, highlighting the need for ongoing vigilance and management.
- UK Biobank will be an invaluable resource for the longitudinal study of RA and related comorbidities, especially as genetic and other results become available.

## Introduction

Rheumatoid arthritis (RA) is a chronic inflammatory syndrome that causes pain, swelling and, if untreated, progressive damage to joints. The UK prevalence of RA has been estimated as 0.8%^1^ which equates to ∼690 000 people. In addition to disability and poorer quality of life, RA is also associated with increased morbidity and mortality compared with the general population.^2^ The leading cause of death among RA patients is cardiovascular disease, with risk 50% higher than the general population.^3^

UK Biobank is a very large general population cohort study of middle-to-older-aged adults in the UK designed to be representative of the general population in terms of age, sex, socioeconomic status and ethnicity. It was created to provide a useful resource to study a wide range of important chronic conditions of adulthood, such as RA. Follow-up is being conducted via linkage to routine administrative data sources such as primary care attendances, hospital admissions, drug prescriptions and death certificates and will, in due course, provide information on incident cases of RA. Genetic, biomarker and imaging data will also become available in future which will greatly enhance the studies that can be undertaken using the UK Biobank cohort. However, because of the age criteria used, large numbers of participants already have prevalent diseases such as RA at recruitment, permitting cross-sectional studies to be undertaken now. It is vital to determine the baseline characteristics of the participants with a specific condition such as RA before subsequent studies can be undertaken and their relevance and significance deduced. The aim of this study was to ascertain the frequency and distribution of reported RA within this very large general population cohort study, and the extent to which it was associated with comorbid conditions. Our work provides a comprehensive baseline evaluation on which many subsequent analyses on this uniquely phenotyped and genotyped cohort (for around n=150 000 and the rest due in Q3 2016) can be performed.

## Methods

### Data collection and definitions

UK Biobank recruited 502 649 participants, aged 37–73 years, between 2006 and 2010. Baseline assessments were conducted at 22 study centres located across the UK.^4^ UK Biobank has generic ethics approval from the North West Multicentre Ethics Research Committee (approval letter dated 17 June 2011, Ref 11/NW/0382). Participants completed touchscreen questionnaires to provide information on sociodemographic factors (including age, sex, ethnicity and postcode of residence), lifestyle (including smoking status and alcohol intake) and medical history (including musculoskeletal, cardiovascular and psychiatric conditions). Postcode of residence is used to derive Townsend scores,^5^ a measure of area-based socioeconomic deprivation. Census data on the percentage of households without a car, overcrowded, not owner occupied and containing unemployed residents are converted into z scores which are summated to provide a Townsend score. The Townsend scores are then used to derive socioeconomic quintiles for the general population. Therefore, the postcode of residence of participants could be used to categorise them into general population socioeconomic deprivation quintiles. Ethnicity was self-reported. Indian, Pakistani and Bangladeshi participants were categorised as South Asian. Physical activity was self-reported and weighted for intensity:^6^ self-reported minutes of walking (×3.3), moderate exercise (×4.0) and vigorous exercise (×8.0). These were then summated to create an overall physical activity score. Study centre staff collected information on current medication and obtained a number of measurements, including height, weight and blood pressure. Participants removed their shoes and heavy outer clothing before weight and height were measured. Weight was measured, to the nearest 0.1 kg, using the Tanita BC-418 MA body composition analyser. Height was measured using a Seca 202 height measure. Body mass index (BMI) was derived from weight (kg)/(height (m)×height (m)). The maximum heart rate during exercise variable is described in detail on the UK Biobank website (http://biobank.ctsu.ox.ac.uk/crystal/field.cgi?id=6033). Briefly, participant ECG readings were taken during cycling and recovery, where the ‘maximum’ score relates to the highest reading taken throughout. Participants self-reported their alcohol intake as ‘never’; ‘special occasions only’; ‘one to three times a month’; ‘once or twice a week’; ‘three or four times a week’; ‘daily or almost daily’. Smoking status was self-reported as ‘never’; ‘past’; ‘current’. Alcohol intake and smoking status were treated as ordinal variables after removing participants who preferred not to answer.

RA was defined as self-report of the condition; UK Biobank did not collect information on disease severity. Therefore, type and ‘intensity’ of medication was used as a proxy measure of valid diagnosis and ‘severity’, stratified into no RA medication; corticosteroids only; one synthetic disease-modifying antirheumatic drug (DMARD) such as methotrexate or sulfasalazine; more than one synthetic DMARD; and one or more biologic DMARD such as anti-tumour necrosis factor (TNF) therapy or rituximab. The full list of eligible drugs recorded by UK Biobank is contained in the online supplementary table S1. We checked the UK Biobank medication list for drugs recorded under trade names; this was rare, but instances are indicated in the table. Participants on combination treatments were grouped according to the highest intensity of medication type based on the aforementioned list. Medications were self-reported.

10.1136/rmdopen-2016-000267.supp1 Supplementary tables

Diabetes, hypertension, cardiovascular disease and depression were based on self-report. Participants self-reported use of lipid/cholesterol-lowering and antihypertensive medication. Participants who did not report hypertension but had measured diastolic blood pressure above 90 or systolic blood pressure above 140 were also categorised as having hypertension. Cardiovascular disease included angina, myocardial infarction, arrhythmias, pericarditis, cardiac failure, valve disease and cardiomyopathy. Participants reported whether they had suffered pain for more than 3 months at a number of sites: no pain, headache, facial, neck or shoulder, back, stomach, hip, knee or pain all over. These data were used to categorise participants into no pain, 1 site, 2–3 sites, 4–7 sites or all over the body.

### Statistical analyses

Participants with and without RA were compared, in terms of their characteristics, lifestyle factors and presence of comorbidity, using χ^2^ tests for categorical data, χ^2^ tests for trend for ordinal data and Kruskal–Wallis tests for continuous data. We used a series of binary logistic regression analyses to examine the association between RA and comorbid conditions adjusted for the potential confounding effects of age, sex, ethnicity and socioeconomic deprivation quintile, as well as potential shared lifestyle risk factors of smoking status, alcohol intake and BMI.

Among those with RA, we used χ^2^ tests for trend to examine whether the characteristics of participants and the prevalence of comorbidity varied according to the type of RA medication, a surrogate marker of RA severity. Participants were excluded from these analyses if their medication was unknown. We undertook generalised ordered logistic regression analyses (GOLOGIT2; http://www3.nd.edu/~rwilliam/gologit2/) to explore the relationship between severity of RA and pain, after adjustment for potential confounders and shared lifestyle risk factors. GOLOGIT2 has the advantage of not assuming a stable relationship between an ordered group variable and outcome.^7^ We used the ‘autofit’ option to relax the parallel-lines constraint where appropriate.

As RA was defined on the basis of self-report, it is possible that some participants with joint symptoms may not have had RA or may have had another musculoskeletal condition that they erroneously labelled as RA. Therefore, we ran the analyses using two definitions of RA. First, we assume that all participants who reported RA had the condition. Second, we used a much tighter definition which included only those participants who reported having RA and were also on relevant medication which rheumatologists would commonly use in RA (see online supplementary table S1). Participants who reported RA but were not recorded as taking relevant medication were excluded from the second analyses. In both sets of analyses, we compared participants with those who did not report RA. We also reran all the analyses excluding the participants who reported osteoarthritis and then also excluding the participants who reported other musculoskeletal conditions, including psoriatic arthritis, systemic lupus erythematosus, ankylosing spondylitis and polymyalgia rheumatic. Finally, we reran the analyses classifying as hypertensive only those participants who reported having the condition. These rerun analyses were not meaningfully different from the final results which are shown. All analyses were conducted using STATA V.13.1 (StataCorp, College Station, Texas, USA).

## Results

### Overall

Of the 502 649 UK Biobank participants, 5657 (1.13%) reported having RA. Of these, 2808 (49.64%) were on no medication; 22 (0.39%) were on steroids only, 1699 (30.03%) were on one synthetic DMARD only, 1009 (17.84%) were on >1 synthetic DMARD; and 119 (2.10%) were on biologics. Therefore, the prevalence of treated RA was 0.57%. The median duration of reported RA was 10 years (IQR 4–20).

Reported RA prevalence was higher in women than men, overall (1.44% vs 0.74%) and by age strata (see online supplementary table S2). Compared with white participants (1.13%), the prevalence was higher among South Asian participants (1.35%), and lower in black (0.90%) and Chinese (0.50%). There was a gradient across the socioeconomic quintiles, from 1.42% in the most deprived participants to 0.99% in the least deprived. Participants with reported RA were more likely to smoke but drank alcohol less frequently (table 1). Those with reported RA had lower physical activity and higher maximum heart rates during exercise (table 2). Participants with RA reported increased use of blood pressure-lowering and cholesterol/lipid-lowering medication (table 1).

**Table 1:** Characteristics of participants with and without rheumatoid arthritis

	No rheumatoid arthritis n=496 992 (98.87%)	All self-reported rheumatoid arthritis n=5657 (1.13%)		Treated rheumatoid arthritis n=2849 (0.57%)	
	n (%)	n (%)	p Value	n (%)	p Value
Sex					
Female	269 516 (54.23)	3951 (69.84)		269 516 (54.23)	
Male	227 476 (45.77)	1706 (30.16)	<0.001	227 476 (45.77)	<0.001
Deprivation quintile					
1 (least deprived)	99 710 (20.09)	998 (17.67)		538 (18.90)	
2	99 171 (19.98)	981 (17.37)		507 (17.81)	
3	99 275 (20.00)	1086 (19.23)		589 (20.70)	
4	99 303 (20.01)	1155 (20.45)		581 (20.41)	
5 (most deprived)	98 916 (19.93)	1427 (25.27)	<0.001	631 (22.17)	0.002
Missing	617	10		3	
Ethnicity					
White	467 503 (96.02)	5327 (96.14)		2716 (97.00)	
South Asian	9755 (2.00)	133 (2.40)		58 (2.07)	
Black	8009 (1.65)	73 (1.32)		22 (0.79)	
Chinese	1593 (0.33)	8 (0.14)	0.004	4 (0.14)	0.001
Missing	10 132	116		49	
Lipid/hypertensive medication use					
Yes	61 156 (87.66)	4228 (75.19)		2103 (73.82)	
No	434 389 (12.34)	1395 (24.81)	<0.001	731 (25.66)	<0.001
Missing	1447	34		15	
Smoking status					
Current	52 278 (10.58)	711 (12.68)		326 (11.53)	
Ex	242 911 (49.17)	2850 (50.84)		1493 (52.81)	
Never	198 854 (40.25)	2045 (36.48)	<0.001	1008 (35.66)	<0.001
Missing	2949	51		22	
Alcohol					
Daily/almost daily	100 983 (20.38)	811 (14.37)		385 (13.53)	
3–4 times/week	114 531 (23.11)	928 (16.44)		412 (14.48)	
1–2 times/week	127 937 (25.82)	1387 (24.57)		750 (26.36)	
1–3 times/month	55 182 (11.14)	689 (12.21)		363 (12.76)	
Occasional	57 074 (11.52)	958 (16.97)		476 (16.73)	
Never	39 794 (8.03)	872 (15.45)	<0.001	459 (16.13)	<0.001
Missing	1491	12		4	

**Table 2:** Characteristics of male and female participants with and without rheumatoid arthritis

	No rheumatoid arthritis n=496 992 (98.87)	All self-reported rheumatoid arthritis n=5657 (1.13)	p Value	Treated rheumatoid arthritis n=2849 (0.57%)	p Value
	Median (IQR)	Median (IQR)		Median (IQR)	
Men					
Age (years)	58 (50–64)	61 (55–65)	<0.001	62 (56–66)	<0.001
Systolic blood pressure (mm Hg)	141 (130–154)	144 (131–157)	<0.001	144 (131–156)	0.005
Diastolic blood pressure (mm Hg)	84 (77–91)	84 (77–91)	0.176	83 (76–90)	0.167
Maximum heart rate during exercise	110 (98–121)	109 (89–121)	0.608	111 (91–125)	>0.999
Physical activity score	2625 (1434–5040)	2946 (1493–5946)	0.014	2610 (1464–5500)	0.541
Body mass index	27.30 (24.98–30.06)	27.97 (25.47–30.85)	<0.001	27.49 (35.07–30.31)	0.353
Waist circumference (cm)	96 (89–103)	99 (91–107)	<0.001	98 (90–106)	<0.001
Waist:hip ratio	0.93 (0.89–0.98)	0.95 (0.91–0.99)	<0.001	0.95 (0.91–0.99)	<0.001
Percentage body fat	25.4 (21.6–29.1)	27 (23.5–31)	<0.001	26.9 (23.2–30.8)	<0.001
Grip strength score	38 (32–44)	32 (22–44)	<0.001	28 (19–36)	<0.001
Women					
Age (years)	57 (50–63)	60 (55–65)	<0.001	61 (55–65)	<0.001
Systolic blood pressure (mm Hg)	135 (122–150)	139 (126–153)	<0.001	140 (127–155)	<0.001
Diastolic blood pressure (mm Hg)	80 (73–88)	81 (74–88)	<0.001	80 (74–88)	0.068
Maximum heart rate during exercise	115 (102–126)	100.5 (88–125)	<0.001	109 (84.5–122)	<0.001
Physical activity score	2514 (1448.5–4533)	2628 (1504–4866)	0.123	244 (1377–4106)	0.120
Body mass index	26.11 (23.45–29.72)	27.22 (24.02–31.29)	<0.001	26.78 (23.75–30.74)	<0.001
Waist circumference (cm)	83 (75–92)	86 (78–96)	<0.001	85 (77–95)	<0.001
Waist:hip ratio	0.81 (0.77–0.86)	0.83 (0.78–0.88)	<0.001	0.83 (0.78–0.88)	<0.001
Percentage body fat	36.8 (32–41.4)	39 (34–43.5)	<0.001	38.7 (33.8–43.4)	<0.001
Grip strength score	22 (18–26)	16 (10–21)	<0.001	12 (8–19)	<0.001
Parity	N (%)	N (%)		N (%)	
Nulliparous	50 458 (18.78)	674 (17.13)		366 (17.79)	
Multiparous	218 243 (81.22)	3261 (82.87)	0.008	1691 (82.21)	0.254
Missing	815	16		4	
Hormone replacement therapy					
No	165 994 (61.94)	1946 (49.68)		1002 (48.90)	
Yes	101 980 (38.06)	1971 (50.32)	<0.001	1047 (51.10)	<0.001
Missing	1542	34		12	
Had menopause					
No	63 651 (23.70)	444 (11.29)		225 (10.94)	
Yes	162 661 (60.58)	2780 (70.67)		1487 (72.32)	
Hysterectomy	30 610 (11.40)	575 (14.62)		283 (13.76)	
Not known	11 599 (4.32)	135 (3.43)	<0.001	61 (2.97)	<0.001
Missing	995	17		5	
Age at menopause (years)					
<50	56 580 (37.21)	1093 (42.09)		567 (40.94)	
50	23 781 (15.64)	386 (14.86)		210 (15.16)	
>50	71 706 (47.15)	1118 (43.05)	<0.001	608 (43.90)	0.015
Missing	117 449	1354		676	

For men and women, we found significant associations between reporting RA and adiposity-related measures—higher BMI, waist circumference, percentage body fat and waist:hip ratio. As additional analysis, we ran linear regressions between RA status and each, adjusted for the covariates of age, sex and smoking and the associations remained. When we used the narrower definition of treated RA and compared this subgroup with participants who did not report RA, all of the associations remained statistically significant, except there was no difference between groups in terms of physical activity score and BMI in men (table 2). Women that reported RA were significantly more likely to report hormone replacement therapy, menopause at all, menopause before the age of 50 and having had a hysterectomy (table 2).

Participants with reported RA had lower grip strength (table 2). They were significantly more likely to report pain, and those with pain were more likely to report it at more than one site or all over the body (table 3). Among participants who reported RA, the number of pain sites increased with increasing RA severity as defined by medication type (table 4). These findings persisted after adjustment for age, sex, ethnicity, socioeconomic quintile, BMI and alcohol intake (table 4). Participants with reported RA had higher systolic blood pressure (table 2). The prevalence of hypertension was higher among those with reported RA (68.04% vs 60.30%; table 3). There was no clear pattern in relation to RA severity based on type of medication (table 5).

**Table 3:** Frequency of comorbid conditions among participants with and without rheumatoid arthritis

	No rheumatoid arthritis n=496 992	All self-reported rheumatoid arthritis n=5657	p Value	Treated rheumatoid arthritis n=2849	p Value
	n (%)	n (%)		n (%)	
Pain					
None	196 103 (39.63)	1049 (18.66)		547 (19.32)	
1 site	136 293 (27.54)	1052 (18.71)		504 (17.80)	
2–3 sites	125 644 (25.39)	1816 (32.30)		887 (31.33)	
4–7 sites	28 644 (5.80)	967 (17.20)		407 (14.38)	
All over body	8107 (1.64)	739 (13.14)	<0.001	486 (17.17)	<0.001
Missing	2159	34		18	
Diabetes					
No	468 450 (94.75)	5173 (92.13)		2635 (92.98)	
Yes	25 966 (5.25)	442 (7.87)	<0.001	199 (7.02)	<0.001
Missing	2576	42		15	
Hypertension					
No	197 330 (39.70)	1808 (31.96)		906 (31.80)	
Yes	299 662 (60.30)	3849 (68.04)	<0.001	1943 (68.20)	<0.001
Missing	0	0			
Cardiovascular disease					
No	467 343 (94.03)	5078 (89.76)		2528 (90.63)	
Yes	29 649 (5.97)	579 (10.24)	<0.001	267 (9.37)	<0.001
Missing	0	0			
Depression					
No	469 172 (94.40)	5263 (93.04)		2680 (94.07)	
Yes	27 820 (5.60)	394 (6.96)	<0.001	169 (5.93)	0.439
Missing	0	0			

χ^2^ for trend for pain; χ^2^ for other variables.

**Table 4:** Generalised ordered logistic regression analysis of the association between rheumatoid arthritis and pain score site

	Pain vs no pain	>1 vs ≤1 site	≥4 vs <4 sites	Whole body vs ≤7 sites
	OR (95% CI)	OR (95% CI)	OR (95% CI)	OR (95% CI)
Univariate				
No RA	1.00	1.00	1.00	1.00
All self-reported RA	2.86*** (2.68 to 3.06)	3.43*** (3.25 to 3.62)	5.42*** (5.12 to 5.75)	9.08*** (8.38 to 9.84)
No RA	1.00	1.00	1.00	1.00
Treated RA	2.74*** (2.50 to 3.01)	3.47*** (3.21 to 3.74)	5.36*** (5.30 to 6.21)	12.44*** (11.26 to 13.75)
No RA	1.00	1.00	1.00	1.00
Steroids only	6.70*** (3.04 to 14.78)	6.70*** (3.04 to 14.78)	6.70*** (3.04 to 14.78)	6.70*** (3.04 to 14.78)
1 synthetic DMARD	2.52*** (2.24 to 2.83)	3.16*** (2.87 to 3.49)	5.35*** (4.82 to 5.93)	11.34*** (9.93 to 12.94)
>1 synthetic DMARD	3.15*** (2.67 to 3.71)	3.92*** (3.44 to 4.47)	6.13*** (5.37 to 7.00)	14.03*** (11.96 to 16.45)
Biologic DMARD	2.89*** (1.82 to 4.60)	4.20*** (2.86 to 6.16)	8.42*** (5.83 to 12.14)	15.16*** (9.69 to 23.74)
Multivariate				
No RA	1.00	1.00	1.00	1.00
All self-reported RA	2.58*** (2.41 to 2.76)	3.04*** (2.87 to 3.22)	4.43*** (4.17 to 4.71)	6.99*** (6.43 to 7.61)
No RA	1.00	1.00	1.00	1.00
Treated RA	2.52*** (2.30 to 2.78)	3.12*** (2.288 to 3.38)	4.86*** (4.46 to 5.28)	10.17*** (9.15 to 11.29)
No RA	1.00	1.00	1.00	1.00
Steroids only	5.32*** (2.36 to 11.96)	5.32*** (2.36 to 11.96)	5.32*** (2.36 to 11.96)	5.32*** (2.36 to 11.96)
1 synthetic DMARD	2.34*** (2.08 to 2.64)	2.86*** (2.59 to 3.17)	4.61*** (4.14 to 5.15)	9.42*** (8.21 to 10.81)
>1 synthetic DMARD	2.84*** (2.41 to 3.36)	3.49*** (3.05 to 3.99)	5.03*** (4.38 to 5.78)	11.19*** (9.45 to 13.25)
Biologic DMARD	2.72*** (1.71 to 4.34)	3.97*** (2.68 to 5.88)	7.34*** (5.02 to 10.72)	12.90*** (8.16 to 20.37)

Multivariate: adjusted for age, sex, ethnicity, deprivation quintile, smoking, body mass index and alcohol intake.

**p<0.01; ***p<0.001.

DMARD, disease-modifying antirheumatic drug; RA, rheumatoid arthritis.

**Table 5:** Frequency of comorbid conditions by severity of rheumatoid arthritis

	Steroids only	1 synthetic DMARD	>1 synthetic DMARD	Biologic DMARD	
	n=22	n=1699	n=1009	n=119	
	n (%)	n (%)	n (%)	n (%)	p Value
Pain					
None	3 (13.64)	349 (20.69)	173 (17.25)	22 (18.49)	
1 site	2 (9.09)	314 (18.61)	171 (17.05)	17 (14.29)	
2–3 sites	10 (45.45)	517 (30.65)	328 (32.70)	32 (26.89)	
4–7 sites	3 (13.64)	239 (14.17)	141 (14.06)	24 (20.17)	
All over body	4 (18.18)	268 (15.89)	190 (18.94)	24 (20.17)	0.150
Missing	0	12	6	0	
Diabetes					
No	21 (95.45)	1574 (92.97)	929 (92.90)	111 (93.28)	
Yes	1 (4.55)	119 (7.03)	71 (7.10)	8 (6.72)	0.972
Missing	0	6	9	0	
Hypertension					
No	5 (22.73)	533 (31.37)	329 (32.61)	49 (32.77)	
Yes	17 (77.27)	1166 (68.63)	680 (67.39)	80 (67.23)	0.721
Missing	0	0	0	0	
Cardiovascular disease					
No	19 (86.36)	1564 (92.05)	866 (87.81)	113 (94.96)	
Yes	3 (13.64)	135 (7.95)	123 (12.19)	6 (5.04)	0.001
Missing	0	0	0	0	
Depression					
No	21 (95.45)	1608 (94.64)	940 (93.16)	111 (93.28)	
Yes	1 (4.55)	91 (5.36)	69 (6.84)	8 (6.72)	0.439
Missing					

χ^2^ for pain; χ^2^ for trend for other variables.

DMARD, disease-modifying antirheumatic drug.

Overall, participants with reported RA had a higher prevalence of diabetes (7.87% vs 5.25%), and this survived correction for variables we considered as confounding (table 3). The prevalence of cardiovascular disease was significantly higher among those with RA, and rates increased with higher severity based on medication except for biologics (table 6). There was a significantly increased rate of depression in participants that reported RA, although this did not persist after adjustment for potential confounders and did not reach statistical significance using the tighter definition of treated RA (table 6).

**Table 6:** Binary logistic regression analysis of the association between rheumatoid arthritis and comorbidity

	Diabetes		Hypertension		Cardiovascular disease		Depression	
	Univariate OR (95% CI)	Multivariate OR (95% CI)	Univariate OR (95% CI)	Multivariate OR (95% CI)	Univariate OR (95% CI)	Multivariate OR (95% CI)	Univariate OR (95% CI)	Multivariate OR (95% CI)
No RA	1.00	1.00	1.00	1.00	1.00	1.00	1.00	1.00
All self-reported RA	1.54*** (1.40 to 1.70)	1.18** (1.06 to 1.32)	1.41*** (1.46 to 1.63)	1.19*** (1.21 to 1.27)	1.80*** (1.65 to 1.96)	1.52*** (1.39 to 1.67)	1.26*** (1.14 to 1.40)	1.07 (0.96 to 1.19)
No RA	1.00	1.00	1.00	1.00	1.00	1.00	1.00	1.00
Treated RA	1.36*** (1.18 to 1.57)	1.16 (1.00 to 1.36)	1.41*** (1.30 to 1.53)	1.28*** (1.17 to 1.39)	1.63*** (1.44 to 1.85)	1.43*** (1.25 to 1.63)	1.06 (0.91 to 1.24)	0.90 (0.77 to 1.06)
No RA	1.00	1.00	1.00	1.00	1.00	1.00	1.00	1.00
Steroids only	0.86 (0.12 to 6.39)	0.63 (0.08 to 4.83)	2.24 (0.83 to 6.07)	1.72 (0.57 to 5.13)	2.49 (0.74 to 8.41)	1.61 (0.45 to 5.70)	0.80 (0.11 to 5.97)	0.75 (0.10 to 5.67)
1 synthetic DMARD	1.36** (1.13 to 1.64)	1.19 (0.98 to 1.46)	1.44*** (1.30 to 1.60)	1.34*** (1.20 to 1.50)	1.36** (1.14 to 1.62)	1.27** (1.06 to 1.53)	0.95 (0.77 to 1.18)	0.81 (0.66 to 1.01)
>1 synthetic DMARD	1.38** (1.08 to 1.76)	1.12 (0.86 to 1.45)	1.36*** (1.19 to 1.55)	1.16* (1.00 to 1.34)	2.19*** (1.81 to 2.64)	1.75*** (1.43 to 2.14)	1.24 (0.97 to 1.58)	1.04 (0.81 to 1.34)
Biologic DMARD	1.30 (0.63 to 2.66)	1.24 (0.59 to 2.63)	1.35 (0.92 to 1.98)	1.38 (0.91 to 2.07)	0.84 (0.37 to 1.90)	0.83 (0.36 to 1.92)	1.22 (0.59 to 2.49)	1.00 (0.48 to 2.06)

Multivariate: adjusted for age, sex, ethnicity, deprivation quintile, smoking, body mass index and alcohol intake.

*p<0.05; **p<0.01; ***p<0.001.

DMARD, disease-modifying antirheumatic drug; RA, rheumatoid arthritis.

The results were very similar when we reran the analyses excluding RA participants who reported other musculoskeletal conditions such as psoriatic arthritis (n=33), systemic lupus erythematosus (n=41), polymyalgia (n=35) and osteoarthritis (n=607), and also when we classified hypertension purely on the basis of self-report. Participants additionally self-reported use of nonsteroidal anti-inflammatory drugs (NSAIDs) and cyclooxygenase-2 (COX-2) inhibitors. We report usage rates in the online supplementary table S3, stratified by RA group. There were significantly increased rates of usage in the narrow and broad RA groups (both p<0.001).

## Discussion

Within UK Biobank, the overall prevalence of RA was 1.13% based on self-report and 0.55% using a narrower definition of treated RA. These are similar to population prevalence estimates based on strict American College of Rheumatology criteria. In a systematic review published in 2006, 19 studies had measured prevalence using these criteria.^8^ Gabriel *et al*^9^ reported a prevalence of 1.1% for the USA, and all the other studies produced a prevalence below 1%. Only two studies have been conducted in the British Isles, and none in the past 12 years. In 1999, Power *et al*^10^ derived a prevalence of 0.5% for Dublin, Ireland, and, in 2002, Symmons *et al*^1^ published a figure of 0.9% for Norfolk, England. Whereas these studies used clinician diagnosis of RA, UK Biobank relies on self-reported diagnosis of RA and use of relevant medication. It should be noted that we could not distinguish between participants that chose not to report (or did not remember) their diseases or medications and those that were genuinely healthy and had nothing to report. We assumed that participants who did not report RA or relevant medication did not in fact have RA; however, this may not necessarily be the case, resulting in a slight underestimation of RA in the sample. In this way, we believe our analysis comparing treated RA with the rest of the population is conservative and therefore robust.

In UK Biobank, the female to male ratio of the prevalence of self-reported RA was 2.3:1. In Alamanos *et al*'s^8^ systematic review, all of the studies demonstrated a higher prevalence in women with the female to male ratio ranging from 1.6:1 to 5.7:1. The only British study to report sex-specific prevalence rates demonstrated a female to male prevalence ratio of 2.5:1, which is very close to our findings in UK Biobank.^1^ Ethnic differences were also observed in UK Biobank. Compared with white participants, the prevalence was higher in South Asian participants and lower in black and Chinese. This is consistent with other studies that have reported a low prevalence among Chinese,^11^ Taiwanese,^12^ South Korean,^13^ black^14^ and black Caribbean communities,^15^ ranging from 0.1 to 0.3 per 100 population in different Asian groups (eg, Far Eastern).^16^

UK Biobank participants who reported RA were more likely to have cardiovascular disease, with an adjusted OR of 1.52 (vs non-RA participants). This finding is consistent with previous studies. Ogdie *et al*^17^ conducted a general population cohort study using routine primary care data. They studied a composite outcome of myocardial infarction, stroke or cardiovascular death among 41 752 participants with RA. In comparison with participants without RA, those with RA were at increased risk irrespective of whether they were taking a DMARD (adjusted HR 1.58, 95% CI 1.46 to 1.70) or not (adjusted HR 1.39, 95% CI 1.28 to 1.50). Similarly, QRISK2 data, which included 531 family practitioners, 2.29 million participants and 140 115 cardiovascular events,^18^ demonstrated that RA was a significant risk factor for stroke among men (OR 1.34, 95% CI 1.19 to 1.51) and women (OR 1.33, 95% CI 1.22 to 1.46). RA was also a risk factor for cardiovascular disease among men (OR 1.38, 95% CI 1.25 to 1.52) and women (OR 1.50, 95% CI 1.39 to 1.61). Hence, UK Biobank data on cardiovascular disease prevalence seem to be in broad agreement with other major datasets, lending external validity. RA was associated with significantly increased rates of NSAID/COX-2 inhibitor use. Interpretation of NSAID use is problematic because these are often taken on a *pro re nata* basis and NSAIDs such as ibuprofen are available as over-the-counter drugs.

It has been suggested that RA and cardiovascular disease may share a genetic predisposition mediated via common inflammatory and metabolic pathways.^19^ ^20^ However, common lifestyle risk factors may also play a role. In a systematic review, Sugiyama *et al*^21^ demonstrated a significant association between current smoking and RA in men (OR 1.87, 95% CI 1.49 to 2.34) and women (1.31, 95% CI 1.12 to 1.54). In our study, participants with RA had a higher prevalence of smoking, and adjustment for potential shared lifestyle risk factors, such as smoking status, somewhat attenuated the association between RA and cardiovascular disease in terms of effect size, suggesting these may explain some of the association.

People with RA have a higher prevalence of some established risk factors for cardiovascular disease, but not others.^22^ Boyer *et al* conducted a meta-analysis of 15 case–control studies, comprising a total of 2956 people with RA; notably in UK Biobank alone, we have nearly the same number of participants in one single cohort and of course multiple number of non-RA participants. Boyer reported a higher prevalence of smoking and diabetes, but not hypertension. We observed a significantly higher prevalence of diabetes in participants that reported RA. UK Biobank will report glycosylated hemoglobin (HbA1c) results on all participants in 2016, and these data will allow better assessment of the association of RA and its severity with glycaemic levels, and whether there is an increased risk of undiagnosed diabetes or impaired glucose tolerance. In contrast to Boyer *et al,* the prevalence of hypertension was significantly higher among UK Biobank participants with RA, and the subgroup on RA medication remained at significantly higher risk of hypertension after adjusting for potential measured confounders. This is similar to what has been reported by other studies.^23^

Overall, there was no association between self-reported RA and depression. This finding is not consistent with previous studies and merits further study. In 2013, Matcham *et al*^24^ published a meta-analysis of 72 studies comprising 13 189 participants. The pooled prevalence of depression was 16.8% (95% CI 10% to 24%), but the estimates of prevalence varied considerably according to case definition up to a maximum of 38.8% based on studies using the Patient Health Questionnaire-9 (PHQ-9^25^). Coexistence of depression and RA is associated with increased pain, fatigue, reduced health-related quality of life, increased physical disability and healthcare costs.^26^ However, depression tends to be underdiagnosed by clinicians. In a cohort study of more than 33 000 patients with RA, depressive symptoms were reported by 11.7% of patients but only identified by 1% of rheumatologists.^27^ Similarly, the incidence of depressive symptoms was 7.8 per 100 patient-years based on patient report, but only 0.4 per 100 patient-years based on rheumatologist reports. The UK Biobank results are based on self-reported depression, and it is possible that people who participate in cohort studies like UK Biobank are unrepresentative of the general population and less likely to feel or report depression.

Of the participants who reported RA, 51% were not on relevant RA medication. Cohorts recruited from secondary care settings^28^ report much higher prevalence (>90%) of DMARD use, but data from inception cohorts of patients presenting with inflammatory arthritis in the community^29^ report lower rates of DMARD usage (<60%), consistent with our findings.

It is not currently possible to formally confirm the diagnosis of RA or stratify RA by disease activity scores, inflammatory markers or self-reported severity in UK Biobank. Therefore, we used type and intensity of RA therapy as a proxy measure of disease severity, on the assumption that more potent RA therapies, such as combination DMARDs or biologic agents, would be used by participants with the most severe disease. This approach could be partially confounded by the more widespread use of treat-to-target strategies resulting in patients with more recently diagnosed RA receiving combination DMARDs earlier than patients with more long-standing disease. Patients who self-reported RA but were on no RA medication are the group in whom confirmation of the diagnosis, via biomarkers, imaging and clinical examination, is most warranted, whereas those on medications are highly likely to have genuine RA. The former group is likely to include people who do not have a true diagnosis of RA (eg, mislabelling of osteoarthritis), but may also include some people with long-standing RA who are unable to take or no longer require DMARD therapy. Among those on RA medications, more potent therapy was associated with a higher prevalence of pain over multiple sites of the body. Conversely, participants on more potent medication were less likely to have cardiovascular disease than those on just steroids. Care needs to be taken in interpreting these findings as genuine negative correlations between disease severity, treatment and comorbidity. First, the number of participants who reported RA but were on steroid medication only was very low (n=12). Second, they may be taking steroids for other indications. Therefore, confirmation of their RA diagnosis via biomarkers and linkage to clinical records is warranted. Third, they may have severe RA but been unable to take more potent therapy due to contraindications, including comorbid conditions. Finally, the association with therapy may reflect reverse causation because patients only continue on these potent but expensive (biologics) and potentially toxic therapies (DMARDs and biologics) if they are responding well to them. Some therapies may also be contraindicated in people with significant existing cardiovascular and other comorbidities. It is also possible that patients with severe RA may decline more potent treatment or may be in a period of remission. The low prevalence of biologic drug treatment in patients with self-reported RA is noteworthy and suggests that the RA population in UK Biobank may be much milder than RA in the UK as a whole; patients with more severe RA appear, as anticipated given the method of population-based recruitment, under-represented in the UK Biobank cohort compared with RA patients in secondary care settings. Patients with less severe disease are more likely to participate in a study like UK Biobank. In terms of identifying the ‘cleanest’ RA phenotype, future studies may consider their primary analysis as treated RA on DMARDs, in the absence of other systematic rheumatic diseases (such as systemic lupus erythematosus (SLE) and osteoarthritis), although there are likely to be patients who do have RA and osteoarthritis, particularly with increasing age. There also exists the possibility of using future biomarker data (especially citrullinated peptide antibody) to better characterise the RA phenotype, as used in other cohorts such as the Women's Health Initiative.^30^

UK Biobank is a very large cohort study that is representative of the general UK population in terms of breakdown by age, sex, ethnicity and socioeconomic status, within the age range recruited. However, in common with similar observational studies, participants are not necessarily representative of the general population in terms of other characteristics, such as lifestyle. Therefore, while UK Biobank is of value in identifying risk factors for the development and progression of disease, care is required in generalising other measures such as disease prevalence. Nonetheless, the prevalence of RA in UK Biobank is broadly in agreement with similar studies, taking into account that case definition is currently based solely on self-report,^31^ and many other characteristics—higher cardiovascular disease prevalence, socioeconomic gradient, more diabetes, pain and lower grip strengths—also match up and extend prior observations. UK Biobank is currently conducting biomarker assays, imaging studies and linkage to clinical records, all of which will improve the case definition and stratification of RA, and therefore the utility of this resource for studying RA. The data reported in this manuscript provide a comprehensive cross-sectional baseline description of RA and relevant comorbidities in UK Biobank. Our results provide an initial analysis from a dataset that is likely to have significant future impactful findings. We did not exhaustively investigate all possible comorbidities at this stage, but instead focused on relatively common psychiatric/cardiometabolic diseases and sociodemographic/anthropometric traits. The follow-up information being collected via linkage to routine administrative data sources, including primary care consultations, hospital admissions, prescriptions and deaths, will provide useful information on the natural history of RA. Finally, ongoing genotyping will enable researchers to explore genetic predisposition, gene–environment interactions and the extent to which the underlying mechanisms are common to other diseases. As these further data become available, UK Biobank will become an increasingly powerful resource to study RA and related comorbidities. We believe this present paper will serve as an excellent starting point for future researchers to interrogate many aspects of RA as UK Biobank data mature.

## References

[1] Symmons DP Epidemiology of rheumatoid arthritis: determinants of onset, persistence and outcome. Best Pract Res Clin Rheumatol 2002;16:707–22. 10.1053/berh.2002.0257 12473269

[2] Young A, Koduri G, Batley M Mortality in rheumatoid arthritis. Increased in the early course of disease, in ischaemic heart disease and in pulmonary fibrosis. Rheumatology (Oxford) 2007;46:350–7. 16908509 10.1093/rheumatology/kel253

[3] Choy E, Ganeshalingam K, Semb AG Cardiovascular risk in rheumatoid arthritis: recent advances in the understanding of the pivotal role of inflammation, risk predictors and the impact of treatment. Rheumatology (Oxford) 2014;53:2143–54. 10.1093/rheumatology/keu224. 24907149 PMC4241890

[4] Allen N, Sudlow C, Downey P UK Biobank: current status and what it means for epidemiology. Heal Policy Technol 2012;1:123–6. 10.1016/j.hlpt.2012.07.003

[5] Townsend P Townsend deprivation index. J Soc Policy 1987;16:126–46.

[6] Celis-Morales CA, Perez-Bravo F, Ibañez L Objective vs. self-reported physical activity and sedentary time: effects of measurement method on relationships with risk biomarkers. PLoS ONE 2012;7:e36345 10.1371/journal.pone.0036345. 22590532 PMC3348936

[7] Williams R Generalized ordered logit/partial proportional odds models for ordinal dependent variables. Stata J 2006;6:58–82.

[8] Alamanos Y, Voulgari PV, Drosos AA Incidence and prevalence of rheumatoid arthritis, based on the 1987 American College of Rheumatology Criteria: a systematic review. Semin Arthritis Rheum 2006;36:182–8. 10.1016/j.semarthrit.2006.08.006 17045630

[9] Gabriel SE, Crowson CS, O'Fallon WM The epidemiology of rheumatoid arthritis in Rochester, Minnesota, 1955–1985. Arthritis Rheum 1999;42:415–20. 10.1002/1529-0131(199904)42:3<415::AID-ANR4>3.0.CO;2-Z 10088762

[10] Power D, Codd M, Ivers L Prevalence of rheumatoid arthritis in Dublin, Ireland: a population based survey. Ir J Med Sci 1999;168:197–200. 10.1007/BF02945853 10540788

[11] Dai SM, Han XH, Zhao DB Prevalence of rheumatic symptoms, rheumatoid arthritis, ankylosing spondylitis, and gout in Shanghai, China: a COPCORD study. J Rheumatol 2003;30:2245–51. 14528524

[12] Kuo CF, Luo SF, See LC . Rheumatoid arthritis prevalence, incidence, and mortality rates: a nationwide population study in Taiwan. Rheumatol Int 2013;33:355–60. 10.1007/s00296-012-2411-7 22451027

[13] Sung YK, Cho SK, Choi CB Korean Observational Study Network for Arthritis (KORONA): establishment of a prospective multicenter cohort for rheumatoid arthritis in South Korea. Semin Arthritis Rheum 2012;41:745–51. 10.1016/j.semarthrit.2011.09.007 22154221

[14] Rasch EK, Hirsch R, Paulose-Ram R Prevalence of rheumatoid arthritis in persons 60 years of age and older in the United States: effect of different methods of case classification. Arthritis Rheum 2003;48:917–26. 10.1002/art.10897 12687533

[15] MacGregor AJ, Bamber S, Silman AJ A comparison of the performance of different methods of disease classification for rheumatoid arthritis. Results of an analysis from a nationwide twin study. J Rheumatol 1994;21:1420–6. 7983640

[16] Alamanos Y, Drosos AA Epidemiology of adult rheumatoid arthritis. Autoimmun Rev 2005;4:130–6. 10.1016/j.autrev.2004.09.002 15823498

[17] Ogdie A, Yu Y, Haynes K Risk of major cardiovascular events in patients with psoriatic arthritis, psoriasis and rheumatoid arthritis: a population-based cohort study. Ann Rheum Dis 2015;74:326–32. 10.1136/annrheumdis-2014-205675 25351522 PMC4341911

[18] Hippisley-Cox J, Coupland C, Vinogradova Y Predicting cardiovascular risk in England and Wales: prospective derivation and validation of QRISK2. BMJ 2008;336:1475–82. 10.1136/bmj.39609.449676.25 18573856 PMC2440904

[19] Rodriguez-Rodriguez L, López-Mejias R, Fernández-Gutiérrez B Rheumatoid arthritis: genetic variants as biomarkers of cardiovascular disease. Curr Pharm Des 2015;21:182–201. 10.2174/1381612820666140825123407 25163740

[20] Sattar N, McCarey DW, Capell H Explaining how ‘High-Grade’ systemic inflammation accelerates vascular risk in rheumatoid arthritis. Circulation 2003;108:2957–63. 10.1161/01.CIR.0000099844.31524.05 14676136

[21] Sugiyama D, Nishimura K, Tamaki K Impact of smoking as a risk factor for developing rheumatoid arthritis: a meta-analysis of observational studies. Ann Rheum Dis 2010;69:70–81. 10.1136/ard.2008.096487 19174392

[22] Boyer JF, Gourraud PA, Cantagrel A Traditional cardiovascular risk factors in rheumatoid arthritis: a meta-analysis. Joint Bone Spine 2011;78:179–83. 10.1016/j.jbspin.2010.07.016 20851020

[23] Manavathongchai S, Bian A, Rho YH Inflammation and hypertension in rheumatoid arthritis. J Rheumatol 2013;40:1806–11. 10.3899/jrheum.130394 23996293 PMC3818311

[24] Matcham F, Rayner L, Steer S The prevalence of depression in rheumatoid arthritis: a systematic review and meta-analysis. Rheumatology (Oxford) 2013;52:2136–48. 10.1093/rheumatology/ket169 24003249 PMC3828510

[25] Kroenke K, Spitzer RL, Williams JB The PHQ-9: validity of a brief depression severity measure. J Gen Intern Med 2001;16:606–13. 10.1046/j.1525-1497.2001.016009606.x 11556941 PMC1495268

[26] van Hoogmoed D, Fransen J, Bleijenberg G Physical and psychosocial correlates of severe fatigue in rheumatoid arthritis. Rheumatology (Oxford) 2010;49:1294–302. 10.1093/rheumatology/keq043 20353956

[27] Rathbun AM, Harrold LR, Reed GW A description of patient- and rheumatologist-reported depression symptoms in an American rheumatoid arthritis registry population. Clin Exp Rheumatol 2014;32:523–32. 24984165

[28] Kiely P, Walsh D, Williams R Outcome in rheumatoid arthritis patients with continued conventional therapy for moderate disease activity-the early RA network (ERAN). Rheumatology (Oxford) 2011;50:926–31. 10.1093/rheumatology/keq406 21169343

[29] Scirè CA, Lunt M, Marshall T Early remission is associated with improved survival in patients with inflammatory polyarthritis: results from the Norfolk Arthritis Register. Ann Rheum Dis 2014;73:1677–82. 10.1136/annrheumdis-2013-203339 23749581 PMC4145442

[30] Mackey RH, Kuller LH, Deane KD Rheumatoid arthritis, anti-cyclic citrullinated peptide positivity, and cardiovascular disease risk in the women's health initiative. Arthritis Rheumatol 2015;67:2311–22. 10.1002/art.39198 25988241 PMC4551571

[31] Aletaha D, Neogi T, Silman AJ 2010 Rheumatoid arthritis classification criteria: an American College of Rheumatology/European League Against Rheumatism collaborative initiative. Arthritis Rheum 2010;62:2569–81. 10.1002/art.27584 20872595

