# Supplementary material for: Characteristics of rheumatoid arthritis and its association with major comorbid conditions: cross-sectional study of 502 649 UK Biobank participants
Source: RMD Open. 2016 Jun 14;2(1):e000267. doi: 10.1136/rmdopen-2016-000267 (PMC4932291; doi:10.1136/rmdopen-2016-000267)
Supplement: Supplementary tables [file rmdopen-2016-000267supp_tables.pdf]

Supplementary Table 1. Medications for rheumatoid arthritis recorded by UK Biobank participants

**Steroids**	**Synthetic DMARDs**	**Biologic DMARDS**
corticosteroids<br>depomedrone<br>triamcinilone<br>methylprednisolone<br>prednisolone<br>prednisone *(also listed in UK Biobank as Deltacortril enteric; Deltastab; Precortisyl; Prednesola)*	Auranofin *(also listed in UK Biobank as Ridaura)*<br>Azathioprine *(also listed in UK Biobank as Imuran)*<br>hydroxychloroquine *(also listed in UK Biobank as Plaquenil)*<br>leflunomide (*also listed in UK Biobank as Arava*)<br>methotrexate<br>methotrexate injections<br>myocrisin<br>penicillamine<br>sulfasalazine *(also listed in UK Biobank as Sulazine; salazopyrin; sulphasalazine)*	abatacept<br>adalimumab *(also listed in UK Biobank as Humira injection solution)*<br>certolizumab<br>etanercept<br>golimumab<br>infliximab<br>rituximab<br>tocilizumab

Supplementary Table 2. Prevalence of rheumatoid arthritis by sex and age

		No rheumatoid arthritis N (%)	Treated rheumatoid arthritis N (%)	Rheumatoid arthritis: all N (%)
**Male**	0-50 years	53,028 (99.64)	84 (0.16)	193 (0.36)
	51-65 years	127,572 (99.25)	433 (0.34)	967 (0.75)
	≥65 years	46,876 (98.85)	271 (0.57)	546 (1.15)
	All ages	227,476 (99.26)	788 (0.34)	1,706 (0.74)
**Female**	0-50 years	64,213 (99.25)	237 (0.37)	482 (0.75)
	51-65 years	157,699 (98.46)	1,283 (0.80)	2,468 (1.54)
	≥65 years	47,604 (97.94)	541 (1.11)	1,001 (2.06)
	All ages	269,516 (98.56)	2,061 (0.75)	3,951 (1.44)
**Both sexes**	0-50 years	117,241 (99.43)	321 (0.27)	675 (0.57)
	51-65 years	285,271 (98.81)	1,716 (0.60)	3,435 (1.19)
	≥65 years	94,480 (98.39)	812 (0.85)	1,547 (1.61)
	All ages	496,992 (98.87)	2,849 (0.57)	5,657 (1.13)

Supplementary Table 3. Self-reported Non-steroidal anti-inflammatory drug (NSAID) and COX-2 inhibitor usage.

		**No rheumatoid arthritis N (%)**	**Treated rheumatoid arthritis N (%)**	**P-value**	**Rheumatoid arthritis: all N (%)**	**P-value**
NSAID use	No	433,325 (87.19)	2,383 (83.64)		4,630 (81.85)	
	Yes	63,667 (12.81)	4766 (16.36)	<0.001	1,027 (18.15)	<0.001
COX-2 inhibitor use	No	496,197 (99.84)	2,751 (96.56)		5,514 (97.47)	
	Yes	795 (0.16)	98 (3.44)	<0.001	143 (2.53)	<0.001

Types of non-steroidal anti-inflammatory drugs (NSAIDs) used included Ibuprofen, ibuprofen plus codeine phosphate, and Naproxen. Types of COX-2 inhibitors examined included Parecoxib, Etoricoxib, Valdecoxib, Rofecoxib and Celecoxib.
